# Getting the metabolites right

**DOI:** 10.7554/eLife.70149

**Published:** 2021-06-15

**Authors:** Arthur Korte

**Affiliations:** Center for Computational and Theoretical Biology, University of WürzburgWürzburgGermany

**Keywords:** *Arabidopsis thaliana*, glucosinolates, specialized metabolites, convergence evolution, parallel evolution

## Abstract

A study of almost 800 *Arabidopsis thaliana* plants from across Europe reveals how the environment and evolutionary pressures shape their metabolites.

**Related research article** Katz E, Li JJ, Jaegle B, Ashkenazy H, Abrahams RS, Bagaza C, Holden S, Pires JC, Angelovici R, Kliebenstein DJ. 2021. Genetic variation, environment and demography intersect to shape Arabidopsis defense metabolite variation across Europe. *eLife* **10**:e67784. doi: 10.7554/eLife.67784

All living organisms look different. Even within a species, individuals can show differences in their shape, size and coloring. This is true both for outward appearances and for physiological traits, such as the concentration of various metabolites. These variations are determined by the underlying genetics of the organism and by the environment it lives in. This results in phenotypic differences that affect the entire organism, allowing it to rapidly respond to changes in its environment. A well-known example of this is the change in color and pattern of butterfly wings under different temperatures, which is regulated by specific hormones (Bhardwaj et al., 2020).

The relationship between genotype and phenotype has been of interest since Mendel postulated the existence of 'internal factors' that are passed on to the next generation (Mendel, 1865), and Bateson originated the term 'genetics' (Bateson, 1909). This link is of major interest in fields ranging from evolutionary biology to molecular biology, and also medicine or agriculture, where untangling genetic effects on phenotypes from environmental effects can lead to beneficial interventions.

The mechanisms by which genetic differences translate into phenotypic differences involve many intermediate steps (Figure 1A); this so-called ‘in-between-ome’ consists of the transcriptome, the proteome and the metabolome. The integration of data from these different levels is necessary to understand how complex phenotypes evolve, and how they are regulated (Subramanian et al., 2020). In plants, the metabolome – all of the small molecules required for an organism to live – is particularly important for adaptation. This is because plants, being immobile, rely on the substances they can absorb from their environment and the derivatives they can produce. However, it is unclear how the variety of metabolites found in plants from different environments arose during evolution. Now, in eLife, Daniel J Kliebenstein, from the University of California Davis, along with colleagues from Austria, the United States and Germany – including Ella Katz as first author – report the factors that drive phenotypic differences in the chemical composition of a specific type of metabolites found in the plant *Arabidopsis thaliana* (Katz et al., 2021).

**Figure 1.**
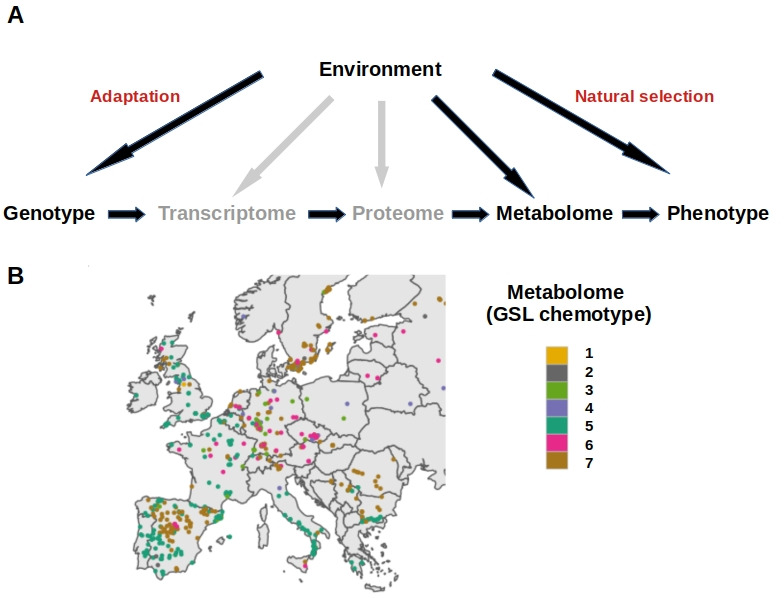
The complex interplay between genotype, phenotype and the environment. (**A**) The environment of an organism influences its phenotype through natural selection, and its genome, which adapts to distinct environments. The mechanisms by which genetic differences translate into phenotypic variation are mediated by the ‘in-between-ome’. This consists of the transcriptome and the proteome, which are indirectly affected by the environment (shown in grey), and the metabolome, which is directly influenced by the environment (shown in black). (**B**) Map of Europe showing dots in different colors representing lines of *A. thaliana* with different glucosinolate profiles (GSL), also known as chemotypes. These chemotypes change depending on the environment, with plants from similar environments exhibiting similar chemotypes.

Katz et al. measured the levels of specialized metabolites called glucosinolates in nearly 800 lines of *A. thaliana* from different ecosystems in Europe. Glucosinolates are biologically active compounds that protect plants from pests and diseases. They are usually found in the Brassicaceae family (Rask et al., 2000), which includes plants like broccoli, cauliflower or mustard, and provide these plants with their characteristic taste. Additionally, plants use glucosinolates to counteract both biotic stress, caused by other living organisms such as herbivores that try to eat the plants, and abiotic stress, such as drought stress (Del Carmen Martínez-Ballesta et al., 2013). The fact that glucosinolates can protect plants from predators, pests and other stressors make it likely that the mechanisms that regulate the abundance of these compounds in individual plants are under strong selection and evolutionary constraints.

The analysis performed by Katz et al. revealed that plants from different regions in Europe had glucosinolates with different chemical compositions or ‘chemotypes’. The spatial distribution of the different chemotypes throughout Europe exhibits distinctive patterns, both at a local and a global scale (Figure 1B). But how did this trait variation evolve? What is its genetic basis and what phenotypic consequences does it have?

To answer these questions, Katz et al. used a technique called genome-wide association mapping (GWAS), which scans the genome of each plant for genetic markers that may be associated with the different chemotypes detected. This analysis found only two major sites in the genome that are responsible for variation in glucosinolate content. However, these previously known loci cannot explain all the variation observed, suggesting that additional loci must also be involved. Additionally, Katz et al. discovered previously unknown alleles at these sites and showed that different combinations of these alleles could lead to similar chemotypes in similar environments.

Based on these observations, Katz et al. suggest that these different combinations of alleles emerged through a mixture of evolutionary events. Some arose through parallel evolution, which occurs when mutations in the same gene lead to similar traits. Others are the result of convergent evolution, where independent mutations in different genes give rise to similar features. Apart from the genetic factors, Katz et al. also show how environmental parameters like climate and geography directly affect the chemotype of *A. thaliana* in different regions. Specifically, they found that specific environmental conditions were associated with certain chemotypes across different geographic locations.

Katz et al. show that many factors, both environmental and genetic, shape the chemotype of glucosinolates in *A. thaliana*. The findings highlight the complexity of the interplay between genetics and the environment, and how both contribute to the evolution of traits in natural populations. They also underline how complex traits appear in a population that is adapted to distinct environments. Additionally, this work demonstrates the need to correctly design studies that aim to explain and dissect complex traits, given that results can be confounded by individuals from the same species being adapted to distinct environmental conditions. Future work will show whether the findings of Katz et al. findings hold for other traits, and if they shed light on general principles of trait evolution.
